# Effects of the dipeptides comprising leucine and lysine on lifespan and age‐related stress in *Caenorhabditis elegans*

**DOI:** 10.1002/fsn3.3256

**Published:** 2023-02-10

**Authors:** Issei Yokoyama, Ou Setoyama, Yaqi Jia, Nana Fujita, Akane Waki, Yusuke Komiya, Jun Nagasao, Keizo Arihara

**Affiliations:** ^1^ School of Veterinary Medicine Kitasato University Towada Japan; ^2^ College of Bioresource Sciences Nihon University Fujisawa Japan; ^3^ Kanagawa Institute of Industrial Science and Technology Ebina Japan

**Keywords:** antiaging, antiglycation, antioxidant activity, bioactive peptide, *Caenorhabditis elegans*, dipeptide

## Abstract

The aging process is affected by various stressors. An increase in oxidative stress is related to the impairment of physiological functions and enhancement of glycative stress. Food‐derived bioactive peptides have various physiological functions, including antioxidant activities. Dipeptides comprising Leu and Lys (LK and KL, respectively) have been isolated from foods; however, their physiological properties remain unclear. In this study, we investigated the antioxidant/antiglycation activity of dipeptides and their antiaging effects using *Caenorhabditis elegans* (*C*. *elegans*). Both dipeptides showed antioxidant activities against several reactive oxygen species (ROS) in vitro. In particular, the scavenging activity of LK against superoxide radicals was higher than KL did. Moreover, dipeptides suppressed advanced glycation end products (AGEs) formation in the BSA–glucose model. In the lifespan assays using wild‐type *C*. *elegans*, both LK and KL significantly prolonged the mean lifespan by 20.9% and 11.7%, respectively. In addition, LK decreased intracellular ROS and superoxide radical levels in *C*. *elegans*. Blue autofluorescence, an indicator of glycation in *C*. *elegans* with age, was also suppressed by LK. These results suggest that dipeptides, notably LK, show an antiaging effect by suppressing oxidative and glycative stress. Our findings suggest that such dipeptides can be used as a novel functional food ingredient. Food‐derived dipeptide Leu–Lys (LK) and Lys–Leu (KL) exert antioxidant and antiglycation activity in vitro. Treatment with LK prolonged the mean lifespan and maximum lifespan of *C*. *elegans* more than that of KL. Intracellular ROS and blue autofluorescence levels (indicator of aging) were suppressed by LK.

## Graphical abstract

Food‐derived dipeptide Leu‐Lys (LK) and Lys‐Leu (KL) exerts antioxidant and antiglycation activity in vitro. Treatment with LK prolonged the mean lifespan and maximum lifespan of *C*. *elegans* than that of KL. Intracellular ROS and blue autofluorescence levels (indicator of aging) were suppressed by LK.

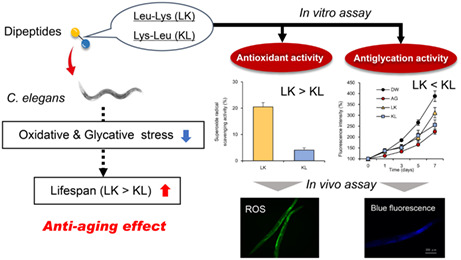

## 1 INTRODUCTION

Aging is a continuous process involving natural changes that occur in most organisms. Changes in tissue and organ functions occur during aging and increase the risk of diseases (e.g., cancer, hypertension, and heart disease). As the incidence of such diseases increases with age (Jaul & Barron, 2017; Niccoli & Partridge, 2012), they are also known as age‐related diseases. Although it is impossible to stop aging, there have been many previous studies on its regulation. The free radical theory, proposed by Herman in 1956, is the most popular mechanism for aging (Harman, 1956; Ziada et al., 2020). Reactive oxygen species (ROS), such as superoxide (O_2_^−^), hydrogen peroxide (H_2_O_2_), and hydroxyl radicals (·OH), are constantly produced from oxygen and scavenged by the antioxidant system in vivo. However, excessive ROS production overwhelms the antioxidant system, causing damage to biomolecules including proteins, lipids, and nucleic acids. This imbalance between ROS production and antioxidant defense is known as oxidative stress (Pizzino et al., 2017). Moreover, an increase in oxidative stress can affect other biological reactions.

Glycation, also known as the Maillard reaction in vivo, is a nonenzymatic browning reaction between reducing sugars (e.g., glucose) and amino compounds (e.g., proteins). This reaction generates numerous chemicals called advanced glycation end products (AGEs), which can interact with ROS production (Tan et al., 2007; Volpe et al., 2018). Although AGEs are essential for the development of color and flavor in processed foods (Arihara et al., 2017; Fu et al., 2020; Losso, 2016), they are frequently used as markers of aging and disease in vivo (Inagi, 2014). Glycative stress refers to a state of high AGEs accumulation in vivo. There is a close relationship between oxidative and glycative stress; therefore, their regulation plays a crucial role in aging.

*Caenorhabditis elegans* is a nonparasitic nematode used as a leading model organism for aging research. The genetic homology between *C*. *elegans* and humans is 60%–80% (Shaye & Greenwald, 2011), which has important significance in applications for human health. Moreover, research using *C*. *elegans* provides insights into several indicators of aging, including lifespan, movement behavior, and nervous system, which can aid in health‐related research (Tissenbaum, 2015; Zhang, Li, et al., 2020). In particular, the short lifespan of *C*. *elegans* (approximately 20 days) is a great advantage in aging research. In the assessment of food safety or functionality, it is urgent to reduce existing animal experiments such as mice and rats (de Boer et al., 2020). Furthermore, the supplementation of antioxidants to *C*. *elegans* prolongs its lifespan and decreases intracellular ROS levels (Kim et al., 2017; Zhang, Li, et al., 2020; Zhang, Zheng, et al., 2020). We also used this model system to elucidate the antiaging effect of Maillard reaction products (Yokoyama et al., 2021). A recent study reported that blue autofluorescence in *C*. *elegans* is derived from AGEs and increases with aging (Komura et al., 2021). Previous reports indicate that the aging process of *C*. *elegans*, as in humans, is also influenced by age‐related stress, and their findings can be applied to evaluate the effects of functional components in foods.

Bioactive peptides derived from food proteins exhibit various physiological properties, such as antioxidant, antihypertensive, and antimicrobial activities. In particular, a large number of studies have focused on the antioxidant activity of peptides (Arihara et al., 2021; Gallego et al., 2019; Stadnik & Keska, 2015). The antiaging effects of bioactive peptides (10–20 amino acids) derived from food proteins have also been investigated in *C*. *elegans* (Yu et al., 2020; Zhou et al., 2018). Recently, Ma et al. (2022) showed that antioxidant peptides (<2 kDa) from dogfish skin significantly extended the lifespan of *C*. *elegans*. However, few studies have examined the effects of small bioactive peptides on the lifespan of *C*. *elegans*. Di/tri‐peptides are absorbed faster than amino acids and proteins (Collin‐Vidal et al., 1994; Hara et al., 1984), suggesting that their effects are exerted rapidly. Furthermore, small peptides can be utilized as food ingredients because their low‐cost production methods have been developed (Shomura et al., 2012; Yokozeki & Hara, 2005). These advantages of small peptides would contribute to develop functional foods and food ingredients.

The specific physiological activities of peptides are based on their unique amino acid composition and sequence. Leu is a hydrophobic amino acid and is frequently contained in the peptide sequence showing antioxidant activity. We previously found that Leu–Lys (LK) and Lys–Leu (KL) exhibited marked antioxidant activity in various synthesized dipeptides comprising Leu (unpublished data). These dipeptides are common sequences in various food proteins. For example, myosin, which is the major protein in skeletal muscle (meat), has three LK and two KL sequences in a part of its heavy chain (300 amino acids). LK has been detected in the traditional Chinese Jinhua ham (approx. 118 μg/g; Zhu et al., 2018). KL has also been isolated from Japanese fermented soybean (approx. 50 μg/g; Sato et al., 2018). Although these dipeptides are ingested from foods, their biological properties and effects on the lifespan of organisms have not been clarified well. This study aimed to evaluate the antioxidant and antiglycation activities of LK and KL in vitro. In addition, we performed a lifespan assay using *C*. *elegans* treated with dipeptides.

## 2 MATERIALS AND METHODS

### 2.1 Chemicals

2′‐Deoxy‐5‐fluorouridine (FUdR), 30% hydrogen peroxide (H_2_O_2_), 4% paraformaldehyde (PFA) phosphate buffer solution, L‐ascorbic acid, bovine serum albumin (BSA), ethylenediaminetetraacetic acid disodium (EDTA‐2Na), FeCl_3_･2H_2_O, glycerin, hypoxanthine, sodium azide, sodium dodecyl sulfate (SDS), and sodium hypochlorite were purchased from FUJIFILM Wako Pure Chemical Co. Carnosine (Car) and xanthine oxidase were purchased from Sigma‐Aldrich. Aminoguanidine hydrochloride, D‐glucose, monopotassium phosphate (KH_2_PO_4_), NaCl, and NaOH were purchased from Kanto Chemical Co. 2‐methyl‐6‐*p*‐methoxyphenylethynyl‐imidazopyrazinone (MPEC) and β‐mercaptoethanol were purchased from ATTO Co. and Nacalai Tesque Inc., respectively. 2′,7′‐dichlorofluorescein diacetate (H_2_DCF‐DA; Invitrogen) and dihydroethidium (DHE; FUJIFILM Wako Pure Chemical Co.) were used for ROS visualization. Synthesized Leu–Lys (LK) and Lys–Leu (KL) were purchased from Scrum Inc.

### 2.2 Evaluation of the antioxidant activity of dipeptides in vitro

#### 2.2.1 DPPH radical‐scavenging activity

The DPPH radical‐scavenging activity of the dipeptides was determined using a previously described method (Ohata et al., 2016) with a slight modification. Briefly, the dipeptides and Car (10 mg/mL) were reacted with DPPH (100 mM) at room temperature (20°C–25°C) for 20 min. Ethanol and Car were used as the control and positive control, respectively. After 20 min, the absorbance of the mixture was measured at 520 nm using a Ultraviolet mini‐1240 spectrophotometer (Shimadzu). The antioxidant activity of the dipeptides against DPPH radicals was calculated using the following formula:

DPPH radical scavenging activity (%) = (absorbance of control − absorbance of peptides/absorbance of control) × 100.

#### 2.2.2 Superoxide radical‐scavenging activity

Superoxide radicals are generated via the hypoxanthine–xanthine oxidase system (Nishikimi, 1975). The scavenging activity against superoxide radicals was measured in accordance with the method proposed in our previous report (Yokoyama et al., 2021). MPEC was reacted with superoxide radicals, and chemiluminescence was measured using Luminescencer‐PSN Ab‐2200 (ATTO Co.). Briefly, 10 μL of dipeptides or Car (10 mg/mL) and 50 μL of 0.72 mM hypoxanthine (0.54 g KH_2_PO_4_, 0.8 g EDTA‐2Na, 0.08 g NaOH, and 0.002 g hypoxanthine) were mixed with 60 μL of xanthine oxidase (0.005 U/mL) and 180 μL of MPEC (300 μM); distilled water (DW) was used as a control. Chemiluminescence of the mixture was measured for 20 s, and the half‐maximal effective concentration (EC_50_) value for each dipeptide solution was determined. The inhibition rate was calculated using the following formula:

Antioxidant activity against superoxide radicals (%) = {(luminescence generated by control − luminescence generated by sample)/luminescence generated by control} × 100.

#### 2.2.3 OH radical‐scavenging activity

Antioxidant activity against OH radicals was evaluated based on the decrease in the protein degradation rate. OH radicals were prepared immediately before performing the protein degradation assay, as previously reported (Ohata et al., 2016). Briefly, 1 mL of 0.13 M H_2_O_2_ was added to 100 μL each of 0.1 M EDTA‐2Na, 100 μL FeCl_3_･2H_2_O, and 0.1 M ascorbic acid. BSA dissolved in saline (0.57 mg/mL) was used as the target protein. Twenty‐five microliters of dipeptides or Car solution (10 mg/mL) was mixed with 175 μL of BSA and incubated for 30 min. OH radicals were reacted with BSA at 37°C for 60 min. The reacted mixture (50 μL) was then added to an equal volume of a sample buffer (1 mL of β‐mercaptoethanol, 5 mL of 0.25 M Tris–HCl, and 0.4 g of SDS). The total volume was then made up to 10 mL with DW, followed by the addition of 10 μL of 70% glycerin. These samples were loaded on a 12.5% gradient SDS‐PAGE gel and electrophoresed at 40 mA for 90 min. The degradation inhibition rates of each peptide solution were calculated using the following formula:

Antioxidant activity against OH radicals (%) = {concentration of BSA in samples (mg/mL)/0.57 (mg/mL)} × 100.

### 2.3 Antiglycation activity in the BSA–glucose model

The antiglycation activity of the dipeptides was evaluated in accordance with a previously described method with slight modifications (Abdelkader et al., 2016). In this study, the specific fluorescence of the AGEs in the BSA–glucose model was leveraged. Briefly, D‐glucose (0.6 M) and BSA (30 mg/mL) were dissolved in 0.15 M phosphate buffer (pH 7.2) containing 0.02% sodium azide as an antibacterial agent. Equal volumes of glucose and BSA were mixed with DW, peptide solutions, or Car (final concentration, 1 mg/mL). Samples in which DW was added instead of BSA were also prepared to exclude AGEs formation between peptides and glucose. Aminoguanidine hydrochloride (final concentration, 1 mg/mL) was used as the positive control. All mixtures were incubated for 7 days at 40°C. The mixture (150 μL) was transferred to a black 96‐well plate (AS ONE Co.). Fluorescence was measured using an Infinite 200 PRO plate reader (Tecan, Männedorf, Switzerland). The excitation (Ex) and emission (Em) wavelengths were 370 nm and 440 nm, respectively. The results were expressed as percentage changes in fluorescence intensity on days 1, 3, 5, and 7 (where incubation equals day 0). The percentage inhibition of AGEs was calculated using the following formula:

AGEs inhibition (%) = {*F*_control_ − (*F*_sample, +BSA_ − *F*_sample, −BSA_)/*F*_control_} × 100.

### 2.4 *C*. *elegans* culture conditions and synchronization

*C*. *elegans* N2 Bristol (wild‐type) was maintained at 20°C in nematode growth medium (NGM) plates seeded with *Escherichia coli* OP50 (Brenner, 1974). Age‐synchronized nematodes were obtained by bleaching gravid adults as described previously (Yokoyama et al., 2021). To obtain synchronized eggs, gravid adults on NGM plates were rinsed with S‐buffer (0.1 M NaCl) and collected in 15 mL tubes. The volume of the solution was adjusted to 4.5 mL using an S‐buffer, after which 500 μL of NaClO (Haiter; KAO) and 100 μL of 10 N NaOH solution were added. The solution containing the nematodes was mixed until the nematode bodies dissolved, and then centrifuged at 4°C and 3000 rpm for 1 min. The precipitate was washed three times with S‐buffer, and the eggs were suspended in 3.5 mL of an S‐buffer. After 24 h, hatched larvae were collected and used for all *C*. *elegans* experiments.

### 2.5 Lifespan assay

To investigate the effect of peptides on lifespan, assays were performed in *C*. *elegans*, following our previous report (Yokoyama et al., 2021). Age‐synchronized L1 stage larvae (approximately 20 worms) were seeded in a liquid medium containing heat‐killed *E*. *coli* OP50. Culture conditions were maintained at 20°C with continuous shaking at 100 rpm for 3 days, and either DW, LK, KL, or Car solution was added (final concentration, 10 mg/mL). Offspring generation was prevented by adding 35 μL of FUdR (final concentration 0.5 mg/mL). The day the test solution was added to the medium was designated as day 0, and worms were cultured further with continuous shaking at 100 rpm. The survival rate was recorded every 3 days until all nematodes had died.

### 2.6 Quantitation of ROS in *C*. *elegans*

The effect of LK on ROS levels was investigated according to previously described methods with minor modifications (Yokoyama et al., 2021). Intracellular ROS levels were measured using the H_2_DCF‐DA reagent. Age‐synchronized L1 stage larvae were seeded in a medium containing *E*. *coli* OP50 and cultured at 20°C for 4 days with continuous shaking at 100 rpm. Adult nematodes were cultured with LK (final concentration, 10 mg/mL) and FUdR for 4 days with continuous shaking at 100 rpm and were washed three times with 300 μL S‐buffer. Subsequently, 500 μL of 50 μM H_2_DCF‐DA was added, and the mixture was incubated for 60 min at room temperature (20°C–25°C) with continuous shaking at 100 rpm. After incubation, the nematodes were fixed in 4% PFA for 10 min and mounted onto 2% agarose pads to obtain a clear image. A fluorescence microscope (BZ‐X800; Keyence) was used to observe *C*. *elegans*. The fluorescence intensity (Ex: 450–490 nm; Em: 500–550 nm) of over 20 worms in each group was analyzed using the ImageJ software (National Institutes of Health).

Dihydroethidium was used to visualize superoxide radicals in *C*. *elegans*. Age‐synchronized L1 stage larvae were seeded in a medium containing *E*. *coli* OP50 and cultured at 20°C for 4 days with continuous shaking at 100 rpm. After 3 days, 35 μL of LK (final concentration, 10 mg/mL) and FUdR were added, and the nematodes were cultured for 4 days with continuous shaking at 100 rpm. DHE was dissolved in S‐buffer to prepare a stock solution (10 mM), which was further diluted in S‐buffer to obtain a 5‐μM working solution. The nematodes were washed thrice with 300 μL of S‐buffer and incubated with 500 μL of DHE solution at 20°C for 30 min with continuous shaking at 100 rpm. Subsequently, nematodes were fixed in 4% PFA for 10 min and mounted onto 2% agarose pads. The fluorescence intensity (Ex: 540–580 nm; Em: 595–670 nm) of over 20 worms in each group was analyzed using the ImageJ software.

### 2.7 Changes in blue autofluorescence with aging

Recently, Komura et al. (2021) reported that an increase in specific autofluorescence is related to AGE formation in *C*. *elegans*. Age‐synchronized L1 stage larvae (approximately 20 worms) were seeded in a liquid medium containing heat‐killed *E*. *coli* OP50. After 4 days, 35 μL of LK solution (final concentration, 10 mg/mL) and FUdR were added to the medium. The day on which LK was added was defined as day 0. Worms were collected on days 3, 10, and 17 and mounted onto 2% agarose pads. The fluorescence intensity (Ex: 340–380 nm; Em: 430–485 nm) of over 20 worms in each group was analyzed using the ImageJ software.

### 2.8 Evaluation of oxidative stress resistance

Age‐synchronized L1 stage larvae were seeded in a medium containing heat‐killed *E*. *coli* OP50 and cultured at 20°C with continuous shaking at 100 rpm for 3 days. Thirty‐five microliters of peptides (final concentration, 10 mg/mL) and FUdR were added, and culturing was continued for 4 days. Each group contained at least 20 worms. Subsequently, adult worms were washed three times with 300 μL of S‐buffer and treated with 25 mM paraquat (Kanto Chemical Co.). Dead worms were counted and recorded every 2 days.

### 2.9 Statistical analysis

All experiments were independently repeated three times. Data are expressed as the mean ± standard error of the mean (SEM). Statistical significance for lifespan differences was analyzed using the log‐rank test. We also conducted one‐ or two‐way repeated ANOVA followed by the Tukey–Kramer multiple comparison test for the measurements of antioxidant and antiglycation activity. Other parameters were analyzed using Student's *t*‐test. All statistical analyses were conducted using Excel‐Toukei ver.7.0 (Social Survey Research Information Co., Ltd.).

## 3 RESULTS

### 3.1 Antioxidant activity of dipeptides

**FIGURE 1:**
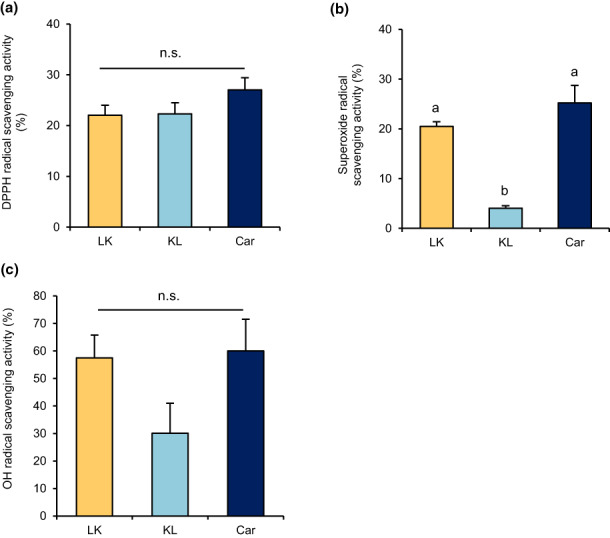
Antioxidant activity of the dipeptides. (a) DPPH radical‐scavenging activity, (b) superoxide radical‐scavenging activity, and (c) hydroxyl radical‐scavenging activity. Data are expressed as the mean of three independent analyses and the SEM. Significant differences were determined by one‐way repeated ANOVA followed by the Tukey–Kramer multiple comparison test (a‐b, *p* < .05). Car, carnosine; KL, Lys−Leu; LK, Leu−Lys.

To determine the antioxidant activity of LK and KL, DPPH, superoxide, and OH/ClO radical‐scavenging assays were performed. Both LK and KL exhibited DPPH radical‐scavenging activity (LK: 22.04% ± 1.96%, KL: 22.31% ± 2.18%, Car: 27.04% ± 2.39% Figure 1a). In contrast, a significant difference in superoxide radical‐scavenging activity was observed between LK and KL (Figure 1b, *p* < .05). LK showed higher scavenging activity (20.49% ± 0.95%) than KL (4.06% ± 0.49%) and the same levels as Car (25.22% ± 3.51%). Moreover, dilutions of LK and KL were used to calculate the EC_50_ values, which were 52.86 mg/mL, >100 mg/mL, and 44.26 mg/mL for LK, KL, and Car, respectively (Table 1). The results of the OH radical‐scavenging activity analysis are shown in Figure 1c. Although there was no significant difference between the OH radical‐scavenging activities, LK also showed higher OH radical‐scavenging activity (57.47% ± 8.32%) than KL (30.10% ± 15.13%). Moreover, the LK scavenging activity against OH radical was also the same as Car (60.04% ± 11.55%).

**TABLE 1:** Effective concentration (EC_50_) of dipeptides against superoxide radical.

	EC50 (mg/mL)
LK	52.86
KL	>100
Car	44.26

Abbreviations: Car, carnosine; KL, Lys−Leu; LK: Leu−Lys.

### 3.2 Antiglycation activity of dipeptides

**FIGURE 2:**
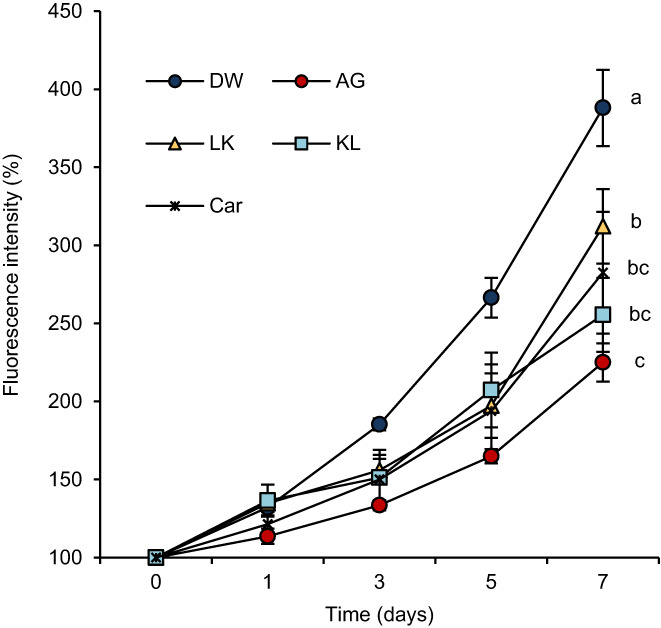
Antiglycation activity of dipeptides in BSA−glucose model. The data are expressed as the mean of three independent analyses and the SEM. Statistical analyses of the fluorescence intensity values were performed by using two‐way repeated ANOVA followed by Tukey–Kramer multiple comparison test. Different letters indicate significant differences on Day 7 (*p* < .05). AG, aminoguanidine hydrochloride; Car, carnosine; DW, distilled water; KL, Lys−Leu; LK, Leu−Lys.

The specific fluorescence intensity of the AGEs increased with incubation time. The addition of dipeptides to the BSA–glucose model significantly inhibited the increase in fluorescence intensity compared with that in the control group (Figure 2). Fluorescence intensity in the control group increased according to incubation time. Significant differences were not observed between control and peptides groups at day 1 (DW: 132.16% ± 0.59%, AG: 113.62% ± 4.92%, LK: 134.72% ± 5.61%, KL: 136.50% ± 10.27%, Car: 121.39% ± 5.71%) and 3 (DW: 185.31% ± 3.95%, AG: 133.48% ± 3.27%, LK: 155.93% ± 7.39%, KL: 151.16% ± 14.67%, Car: 150.05% ± 18.86%). However, the fluorescence intensity of peptides groups was suppressed at day 5 (DW: 266.46% ± 12.70%, AG: 164.92% ± 4.52%, LK: 197.28% ± 20.66%, KL: 207.24% ± 23.93%, Car: 193.87% ± 29.88%). On day 7, KL showed higher inhibitory activity than LK, which was equivalent to that of the positive control on day 7 (DW: 388.04% ± 24.40%, AG: 224.96% ± 12.24%, LK: 312.14% ± 23.79%, KL: 255.43% ± 23.70%, Car: 282.49% ± 38.98%, *p* < .05).

### 3.3 Effects of dipeptides on the lifespan of *C*. *elegans*

**FIGURE 3:**
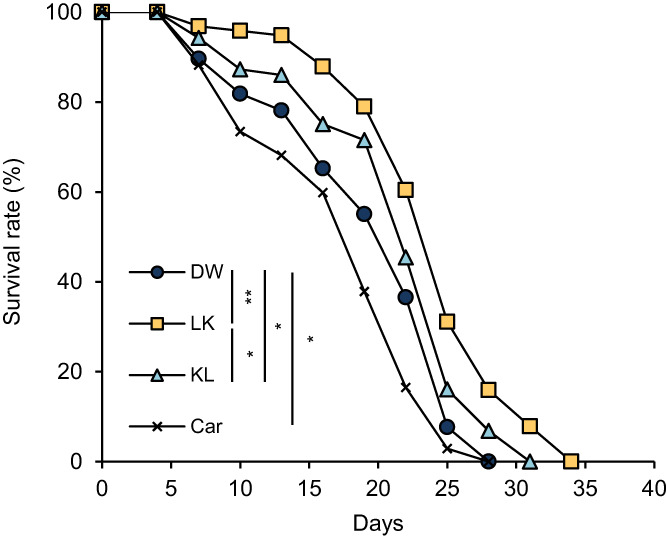
Effect of dipeptides on the lifespan of *C*. *elegans*. The changes in survival rate are expressed as the mean of three independent analyses. Statistical analysis of the differences between the control groups was performed using the log‐rank test. *p < .05, **p < .01. Car, carnosine; DW, distilled water; KL, Lys−Leu; LK, Leu−Lys.

**FIGURE 4:**
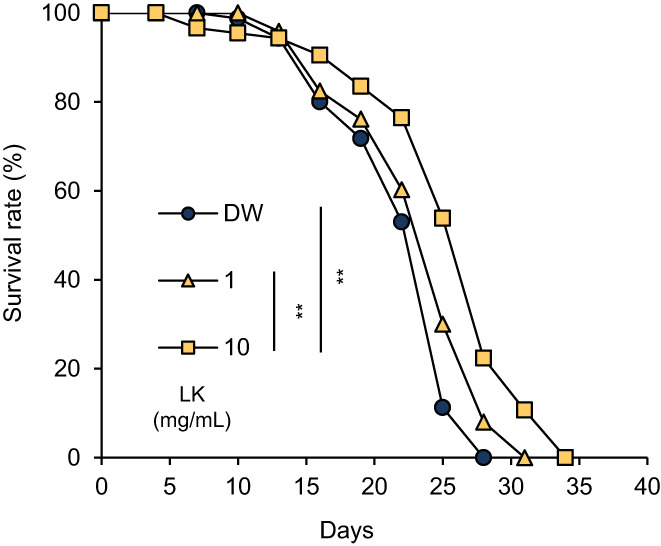
Dose‐dependent effect of LK treatment on the lifespan of *C*. *elegans*. The changes in survival rate are expressed as the mean of three independent analyses. Statistical analysis of the differences between the control groups was performed using the log‐rank test. **p < .01. DW, distilled water; LK, Leu−Lys.

Based on the in vitro results, we measured the lifespan of *C*. *elegans* treated with the dipeptides. Both LK and KL significantly prolonged the lifespan of *C*. *elegans*, and the duration of the treatment with LK was longer than that of the treatment with KL (Figure 3). As shown in Table 2, the mean lifespan (control: 20.52 ± 0.72 days, LK: 24.81 ± 0.61 days, KL: 22.92 ± 0.68 days, Car: 18.16 ± 0.71 days) and the maximum lifespan (control: 26.33 ± 1.67 days, LK: 34.00 ± 1.73 days, KL: 31.00 ± 1.73 days, Car: 26.20 ± 1.20 days) were affected by treatment with the dipeptides. However, the treatment with Car significantly shortened the mean lifespan of *C*. *elegans*. Compared with the control group, the mean lifespan of *C*. *elegans* was significantly prolonged by 20.91% (*p* < .01) and 11.70% (*p* < .05) after treatment with LK and KL, respectively. A significant difference was also observed between dipeptides (*p* < .05). In addition, the assay was performed by adding LK solution at final concentrations of 1 and 10 mg/mL (Figure 4 & Table 3). LK of 10 mg/mL showed a strong life extension effect compared with 1 mg/mL LK (*p* < .05). The results of this test showed that the degree of lifespan extension by LK increased in a dose‐dependent manner.

**TABLE 2:** Effect of dipeptides on the lifespan in *C*. *elegans*.

Group	Total worms	Mean lifespan (day)	% of control	Maximum lifespan (day)
Control	101	20.52±0.72a	–	26.33±1.67a
LK	127	24.81±0.61b	+20.91	34.00±1.73b
KL	109	22.92±0.68c	+11.70	31.00±1.73c
Car	115	18.16±0.71d	−11.14	26.20±1.20a

*Note*: Different letters indicate significant differences (*p* < .05).

Abbreviations: Car, carnosine; KL, Lys−Leu; LK: Leu−Lys.

**TABLE 3:** Effect of LK concentration on the lifespan in *C*. *elegans*.

Group		Total worms	Mean lifespan (day)	% of control	Maximum lifespan (day)
	Control	119	22.31±0.72a	–	28.00±0.11a
LK (mg/mL)	1	115	23.03±0.61a	+3.2	29.25±0.74b
	10	120	26.08±0.68b	+16.9	33.20±0.73c

*Note*: Different letters indicate significant differences (*p* < .05).

Abbreviation: LK, Leu‐Lys.

### 3.4 Effect of LK on ROS and glycation levels in *C*. *elegans*

**FIGURE 5:**
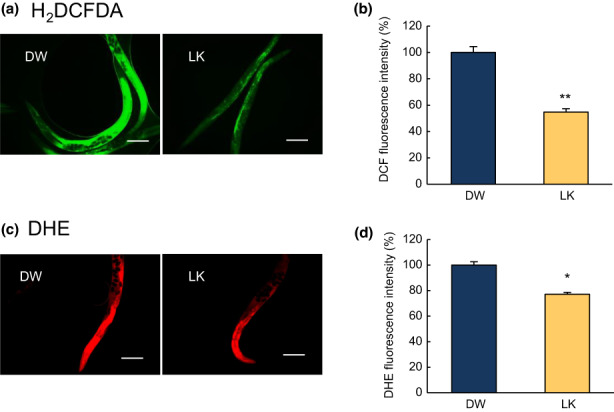
Effects of LK treatment on the intracellular ROS and superoxide radical levels. (a) Images of H_2_DFCDA in *C*. *elegans*; (b) Quantification of the fluorescence to determine intracellular ROS levels. (c) Images of DHE staining in *C*. *elegans*; (d) Quantification of the fluorescence to determine superoxide radical levels. Scale bar: 200 µm. The data are expressed as the mean of three independent analyses and the SEM. Statistical analysis of the differences between the control groups was performed using Student's *t*‐test. *p < .05, **p < .01. LK, Leu−Lys.

**FIGURE 6:**
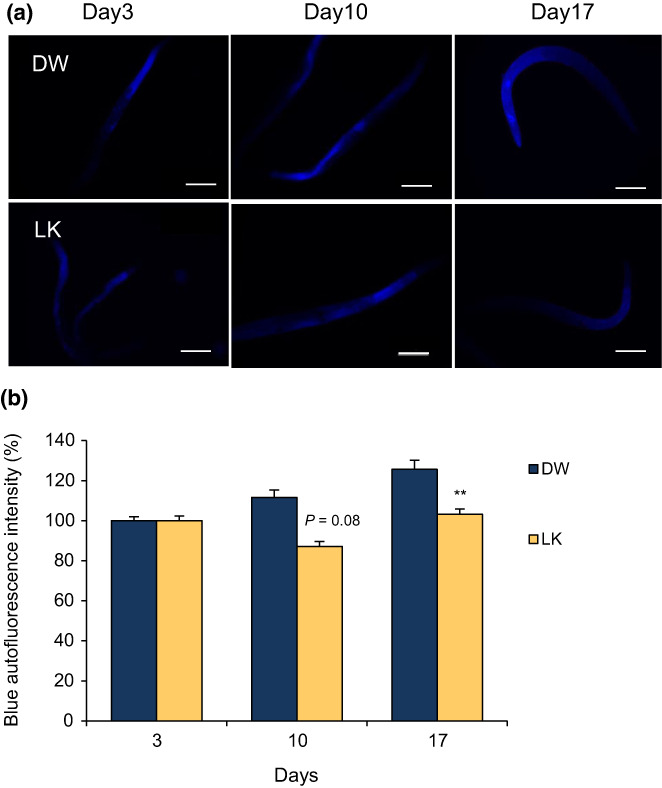
Measurement of blue autofluorescence intensity with age. (a) Images of blue autofluorescence in *C*. *elegans*; (b) Quantification of fluorescence intensity. Scale bar: 200 µm. The data are expressed as the mean of three independent analyses and the SEM. Statistical analysis of differences between the control groups was performed by using two‐way repeated ANOVA followed by Student's *t*‐test. *p < .05, **p < .01. LK, Leu‐Lys.

We evaluated the effect of LK treatment on oxidative and glycative stress in *C*. *elegans*. H_2_DCF‐DA generates fluorescent substances through oxidation by various ROS, such as H_2_O_2_, OH radicals, and peroxynitrite radicals (Kalyanaraman et al., 2012). As shown in Figure 5a, compared with those in the control group, intracellular ROS levels were significantly decreased by 40% in nematodes treated with LK (Figure 5b, *p* < .01). Subsequently, DHE staining was performed to determine the effect of LK on the superoxide radical levels in *C*. *elegans*. LK treatment also significantly decreased by 20% (Figure 5c,d, *p* < .05). These results suggest that LK acts as an antioxidant, both in vitro and in vivo. Next, we investigated whether LK exerted antiglycation activity in vivo. The intensity of blue autofluorescence increased with aging in the control group. In the LK treatment group, the blue autofluorescence intensity was suppressed on day 17 (*p* < .05, Figure 6a,b). This result suggests that the antiglycation activity of LK could also be exerted in vivo.

### 3.5 Effect of LK on the lifespan of *C*. *elegans* under oxidative stress

**FIGURE 7:**
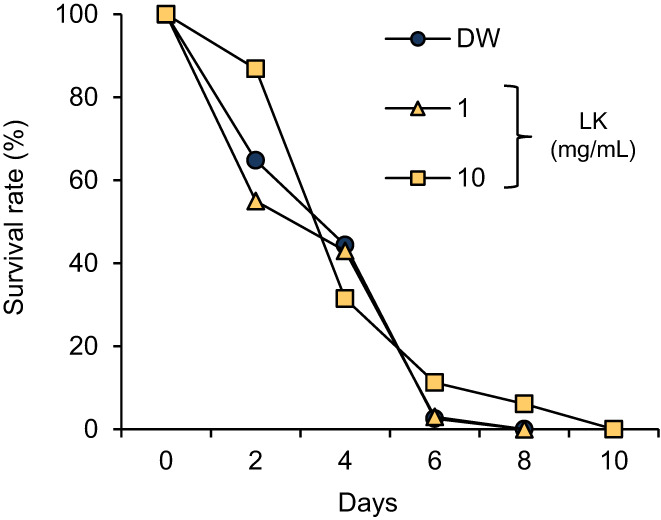
Effect of LK treatment on the lifespan of *C*. *elegans* under oxidative stress. The changes in survival rate are expressed as the mean of three independent analyses. DW, distilled water; LK, Leu−Lys.

Changes in signaling pathways alter the expression of genes associated with antioxidant potential and stress response, resulting in the longevity of *C*. *elegans*. Since the exposure of *C*. *elegans* to paraquat induces an increase in ROS levels, it is frequently used to investigate oxidative stress tolerance (Yokoyama et al., 2021; Zhang, Li, et al., 2020; Zhang, Zheng, et al., 2020). Nematodes treated with LK were exposed to paraquat; however, the effects of LK on the mean lifespan were not observed (Control: 4.32 ± 0.30 days, 1 mg/mL: 4.20 ± 0.07 days, 10 mg/mL: 4.25 ± 0.19 days; Figure 7 &Table 4).

**TABLE 4:** Effect of LK on the lifespan under oxidative stress.

	Group	Total worms	Mean lifespan (day)	Maximum lifespan (day)
Oxidative stress (25 mM paraquat)				
	Control	90	4.32±0.30a	7.33±0.77a
LK (mg/mL)	1	80	4.20±0.07a	7.00±0.19a
	10	88	4.25±0.19a	7.33±0.45a

Abbreviation: LK, Leu−Lys.

## 4 DISCUSSION

Food‐derived bioactive peptides exhibiting antioxidant, antihypertensive, and immunomodulating effect have been reported. Such bioactive peptides are also utilized as a functional food ingredient. Carnosine and anserine, typical antioxidant dipeptides in meats and fish, also exert antioxidant activity against OH radicals. However, their activity against DPPH radicals is extremely low (Abdelkader et al., 2016; Terashima et al., 2007). Other dipeptides derived from foods also exhibit scavenging activity against superoxide radical but not DPPH radical (Ozawa et al., 2022). Moreover, Yanai et al. (2008) reported the existence of different mechanisms underlying the radical‐scavenging properties of antioxidants. Our results suggested that the antioxidant activities of LK and KL are more effective against ROS such as superoxide radicals than against DPPH radicals. The reason for such results includes the sequence of peptides. For example, hydrophobic amino acids (e.g., Ala, Val, and Leu) at N terminus relate to an increase in antioxidant activity (Sabeena Farvin et al., 2010). LK also contains Leu at the N terminus; therefore, it seems to possess a higher antioxidant activity than KL.

Dipeptides showed antiglycation activities by suppressing AGEs fluorescence intensity in the BSA−glucose model. Aminoguanidine hydrochloride, used as a positive control, exhibits antiglycation activity by trapping carbonyl compounds (Abbas et al., 2016; Nagai et al., 2012). Among dipeptides, Asn‐Trp derived from yam hydrolysis and carnosine showed antiglycation activity in vitro (Freund et al., 2018; Han et al., 2014). Such antiglycation substrates inhibit AGEs formation by reacting with carbonyl compounds, which would be related to the Maillard reaction degree in vitro. Therefore, we confirmed the reactivity of each dipeptide with glucose and found that the reactivity of KL was three times higher than that of LK (Figure S1). Similarly, it has also been shown that the reactivity between KL, among all dipeptides containing Lys at the N terminus, and glucose is the highest (Liang et al., 2016). Depending on model conditions, Lys is one of the more reactive amino acids in the Maillard reaction. Our results, along with those of a previous report, suggest that the antiglycation activity is influenced by peptide sequence.

Between the two dipeptides used in this study, one showed high antioxidant activity (LK), and the other showed superior antiglycation activity (KL). However, the effect on the lifespan was greater in the LK group. Therefore, it was assumed that the effects of the antioxidant activity of the dipeptides on the longevity of *C*. *elegans* were more prominent than their antiglycation activities. According to the result of lifespan assay, we used only LK for further experiments. However, there is a possibility that the antioxidant activity of KL would be increased by the Maillard reaction in vitro. Maillard reaction products exhibit strong antioxidant activity and increase the lifespan of *C*. *elegans* (Papaevgeniou et al., 2019; Yokoyama et al., 2021). Since KL has high reactivity in the Maillard reaction, its Maillard reaction products may affect the longevity in *C*. *elegans*. Carnosine, which exhibited antioxidant and antiglycation activity, unexpectedly shortened the mean lifespan of *C*. *elegans*. Kingsley et al. (2021) also reported that carnosine treatment under normal conditions did not affect the mean lifespan of *C*. *elegans*, but rather shortened at high concentration. Unlike LK and KL, carnosine may be harmful to *C*. *elegans* at a concentration of 10 mg/mL.

In *C*. *elegans*, blue autofluorescence has been measured as an indicator of age‐related pigment (lipofuscin) (Wang et al., 2018; Zhao et al., 2021; Zhou et al., 2018). Moreover, it has shown that this fluorescence relates to AGEs accumulation, and is reduced by antioxidants (Komura et al., 2021). In our results, LK also decreased intracellular ROS levels and blue autofluorescence intensity in *C*. *elegans*. Thus, reduction in oxidants by antioxidants such as LK lead to a decrease in blue autofluorescence. Natural foods, such as commonly consumed fruits, also decrease ROS and autofluorescence levels, inducing longevity in *C*. *elegans* (Carlsen et al., 2010; Navarro‐Hortal et al., 2022; Vayndorf et al., 2013). Therefore, the ingestion of LK and KL may have positive benefits in other organisms, such as humans. Although large doses are required when taken from food, this can be compensated for by using it as a food additive. Furthermore, no toxicity was observed with 10 mg/mL of peptides in our study. However, it should not be determined whether 10 mg/mL is the effective concentration in humans; therefore, further studies are needed.

A dipeptide Tyr‐Ala derived from maize protein exerts antioxidant activity and prolongs the lifespan in *C*. *elegans* (Zhang et al., 2016). In this previous study, the life extension effect did not change regardless of *E*. *coli* condition (live/killed). We also examined the effect of live *E*. *coli* on the lifespan extension of *C*. *elegans*. Although the lifespan was only prolonged by LK treatment, its effect was reduced by half compared with that of heat‐killed *E*. *coli* (Figure S2 & Table S1). Edwards et al. (2015) also used heat‐killed *E*. *coli* to avoid catabolism in a lifespan assay with amino acids. LK may be decomposed by live *E*. *coli* and its life extension effect changes through catabolism. This change indicates that the functional properties of LK might affect lifespan extension.

Several signaling pathways influence the longevity of *C*. *elegans* (Lapierre & Hansen, 2008). The insulin/insulin‐like growth factor‐1 (Ins/IGF‐1) signaling pathway, which is conserved in flies, mice, and humans, modulates various cellular processes, such as metabolism and stress response. The target of the rapamycin (TOR) pathway also regulates the metabolic response and degree of aging via a nutrient sensor. Blueberry extracts contribute to the longevity of *C*. *elegans* through Ins/IGF‐1 signaling pathway (Wang et al., 2018). Dipeptide Tyr‐Ala also enhances oxidative stress tolerance via the Ins/IGF‐1 signaling pathway in *C*. *elegans* (Zhang et al., 2016). However, LK did not prolong the lifespan in *C*. *elegans* treated with paraquat in this study. Signal changes induce the upregulation of genes related to stress defense and then obtain resistance to various stress. Dose‐dependent changes in mRNA expression through signaling pathways have also been reported (Wang et al., 2018; Zhang, Li, et al., 2020; Zhang, Zheng, et al., 2020). We assume that this may be caused by the difference in the functional potentials of samples. In previous study, 1 mM (≒ 0.27 mg/mL) of Tyr‐Ala significantly prolonged the lifespan under oxidative stress, but LK did not even 10 mg/mL. Since peptide sequence affects its functionality including antioxidant activity, it may also influence the change in signaling pathway. To elucidate the detailed relationship between LK and signaling pathway, the comparison with various peptides and the assay using mutant worm are needed. Our results, along with those of a previous study, suggest that 10 mg/mL of LK would contribute to longevity without changes in the signaling pathways.

## 5 CONCLUSION

Aging is observed in all animal species. During aging process, stresses related to oxidation and glycation accumulate naturally. Previous studies have reported that food‐derived bioactive peptides exert various physiological effects, such as antioxidant effects. In this study, we found that the synthesized dipeptide LK and KL exhibited antioxidant and antiglycation activity. It was assumed that their activities are affected by peptide sequence. Both dipeptides significantly prolonged the lifespan of *C*. *elegans*, but LK exerted more effect than KL. Moreover, treatment of *C*. *elegans* with LK suppressed ROS and blue autofluorescence. Thus, LK may affect longevity by decreasing age‐related stress. These findings suggest that dipeptides can be utilized as a functional food ingredient for health promotion.

## CONFLICT OF INTEREST STATEMENT

The authors declare that they have no conflict of interest.

## ETHICAL APPROVAL

This study does not involve human or animal experiments.

## Supporting information


Appendix S1

## Data Availability

The data that support the findings of this study are available from the corresponding author upon reasonable request.
