# Supplementary material for: Effects of the dipeptides comprising leucine and lysine on lifespan and age‐related stress in Caenorhabditis elegans
Source: Food Sci Nutr. 2023 Feb 10;11(6):2776–86. doi: 10.1002/fsn3.3256 (PMC10261761; doi:10.1002/fsn3.3256)
Supplement: Supplementary file 1 — Appendix S1 [file FSN3-11-2776-s001.docx]

**Table S1** Effect of dipeptide on the lifespan in *C. elegans* fed live-*E. coli*.

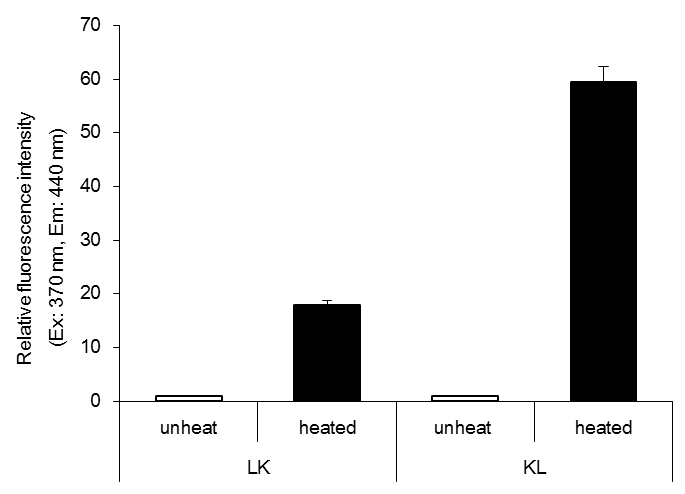


**Figure S1**. Comparison of the Maillard reaction degree in dipeptides-glucose systems

The solutions of dipeptide (10 mg/mL) and glucose (50 mM) were prepared in 0.25% sodium carbonate buffer, and mixed. Each mixture was heated at 90℃ for 2h in the dry thermos unit. After heat treatment, mixtures were cooled and measured its fluorescence intensity (Ex: 370 nm, Em: 440 nm). Data are expressed the mean ± standard error.


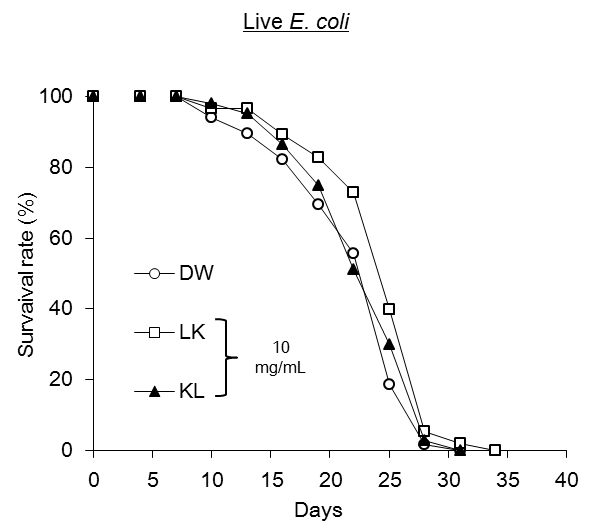


**Figure S2**. Effect of dipeptide on the lifespan in *C. elegans* fed live-*E. coli*
